# Pharmacogenetics of cancer therapy: breakthroughs from beyond?

**DOI:** 10.4155/fso.15.80

**Published:** 2015-11-01

**Authors:** Da-Yong Lu, Ting-Ren Lu, Bin Xu, Jian Ding

**Affiliations:** 1 School of Life Sciences, Shanghai University, Shanghai 200444, PR China; 2 Shanghai Institute of Materia Medica, Chinese Academy of Sciences, Shanghai 201203, PR China

**Keywords:** antineoplastic drugs, cancer stem cells, cost–effectiveness study, drug targets, drug toxicity, neoplasm metastasis, personalized cancer therapy, pharmacogenetics

## Abstract

‘Pharmacogenetics or Pharmacogenomics’ (PG) is one of the most practiced cancer therapeutic strategies, tailored for individualized patients. Despite its popularity and rapid advancements in the field, many obstacles for cancer therapy PG still need to be overcome. By borrowing scientific systems from other disciplines such as cancer diagnosis, and therapeutic information from the diversity of tumor origins, categories and stages, cancer therapy PG may hopefully be improved. Furthermore, to quickly acquire genetic and pathologic information and seek therapeutic interventions, possible breakthroughs may come from beyond – changing the cancer therapeutic landscapes. The next generations of PG protocols and hospital routines for searching deadly cancer pathogenic pathways versus drug-targeting predictions are of great clinical significance for the future. Yet, progress of cancer therapy PG is entering into a bottleneck stage owing to simple model of relevant techniques and routines. Promoting or even innovating present PG modular is very necessary. This perspective highlights this issue by introducing new initiatives and ideas.

**Figure 1.**
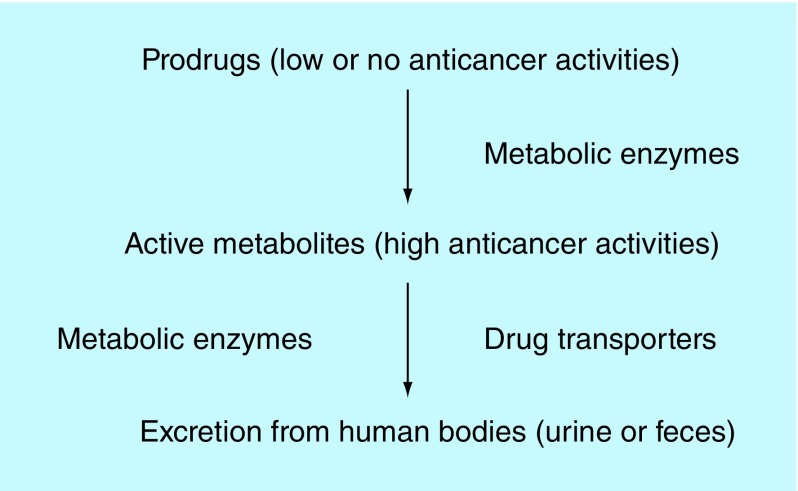
**Outlook of anticancer prodrugs in human bodies.**

**Figure 2.**
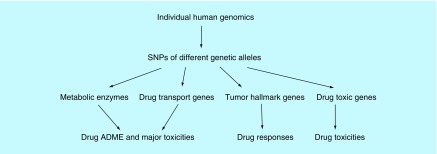
**Outlook of anticancer pharmacogenetics.** ADME: Drug absorption, distribution, metabolism and excretion; SNP: Single nucleotide polymorphism.

**Figure 3.**
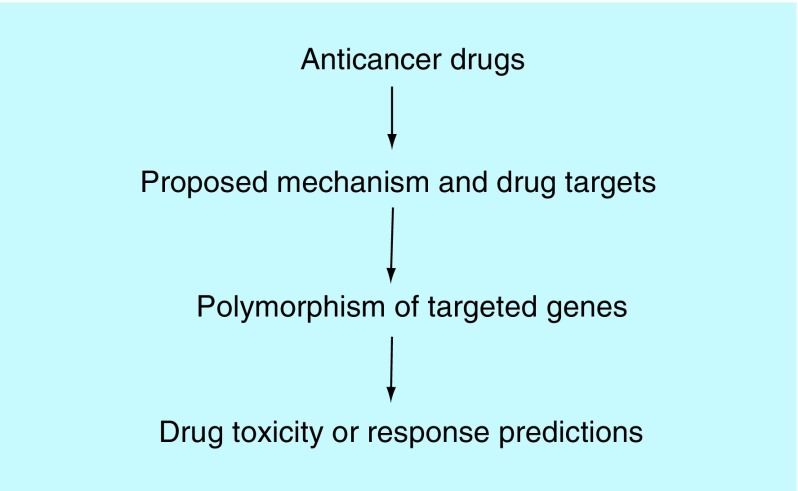
**Pharmacogenetics outlook of drug-oriented drug toxicity and efficacies predictions.**

**Figure 4.**
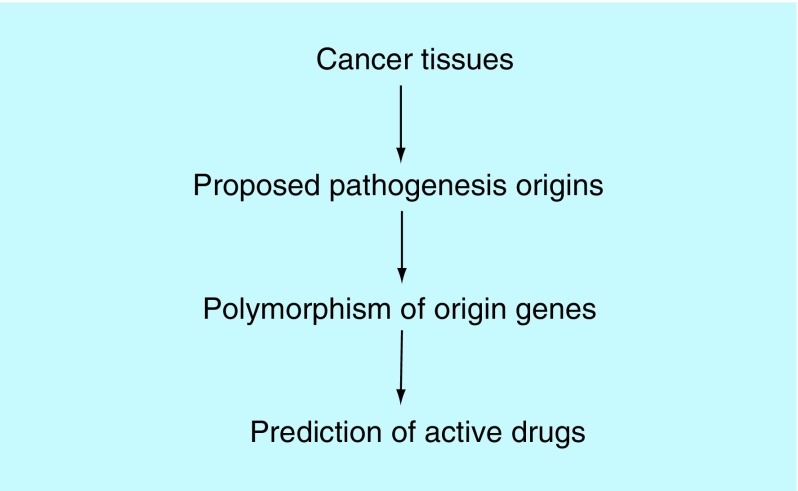
**Pharmacogenetics outlook of cancer pathogenesis-oriented drug and combination selecting.**

**Figure 5.**
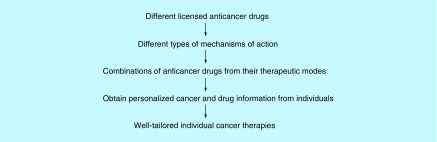
**Outlook of drug combinations and individualized cancer therapies.**

## Background

Cancer is a common disease that claims the lives of about 7–10 million people annually across the world. As a result, cancer remains a great medical challenge worldwide [1,2]. Many efforts can impact the overall therapeutic efficacies and outcomes of cancer treatments. One of these efforts is personalized cancer therapy. Pharmacogenetics (PG), one of the clinical personalized strategies has been developing into a general modular of detecting genetic polymorphisms for seeking optimal therapeutic interventions in individual cancer patients and a few fruitful outcomes have been achieved in the past several decades. Cancer therapy PG has become one of the most important frontiers of personalized cancer therapeutics worldwide [3–11]. In the initial paradigms of PG, efforts are focusing on maximizing therapeutic efficacy and minimizing drug toxicities in patients by detecting patient's genetics of metabolizing enzymes, which are recognized as branches of pharmacokinetics or pharmacodynamics. Drug absorption, distribution, metabolism and excretion (ADME) are the main themes of these studies [11]. More recently, cancer therapy PG is also emphasizing drug response or toxic-related pathways or pathogenesis links by detecting polymorphisms of drug targeting or toxic genes, proteins, growth factors and/or other dysfunction molecules. Upcoming sections will discuss and highlight these issues.

Despite the popularity of cancer therapy PG, human genetic information used for forecasting disease risk, therapeutic agent options, drug characteristics (doses/toxicities and responses to cancer) in individual humans have not been perfected yet. The similarities and differences of PG between cancer therapy and other disease therapies are important for future scientific investigations and therapeutic improvements. Possible future perfections are proposed herein.

## Current cancer therapy PG

### Drug ADME & genetics of metabolic enzymes in individual humans & patients

Drug ADME studies by polymorphism analysis of individual metabolic enzymes and approximately 300 human metabolic enzyme genes and molecules have been subjected to PG investigations and clinical applications [11]. Technically, no major difference between anticancer drug PG and other disease therapeutic PG systems has been designed and clinically applied. However, a growing number of prodrugs have been entering into markets, such as Irinotecan, Topotecan and1 MST-16 etc., in cancer treatments [3,4,8–10]. For these prodrugs, the polymorphisms of drug-metabolizing enzymes (DME) are important parameters for predicting the rate of active metabolites in the cancer patient blood, general organ or tumor tissue accumulations. DME-relative molecules are mainly different isoforms of cytochrome P450 monoxygenases (CYP) (>70 CYP enzyme isoforms) and serviced for major genotyping of human DME; if only a small amount of active metabolites is transformed from prodrugs by polymorphism-induced loss activity of DME, the upcoming therapeutic efficacies can be less effective and indecisive. If too much active metabolites of drugs are produced, the high therapeutic toxicity or even secondary tumors will be possible. Mixed characteristics of prodrug responses and severity of toxicities can randomly happen in many cancer patients following prescriptions of normal doses of anticancer drugs or prodrugs, especially cytotoxic anticancer drug treatments. More recently, ADME analysis for prodrug and epidrugs is being emphasized in cancer therapy PG [8–10]. Besides prodrugs, ADME for normal anticancer drugs is also the first choice at the present time.

### Drug targets & response predictions by tumor refractory & metastatic-related pathways

The paramount task of greatest therapeutic significance is to find the biologic relationships between disease progression (tumor genetic mutations/invasive/remote metastasis) and therapeutic outcomes (relevant anticancer drugs selections and applications). Ninety percent of cancer deaths are caused by neoplasm metastasis [12–16] and cancer stem cells [17–22] that then have been proposed to contribute to tumor relapse, metastasis and therapeutic resistance and so on. PG for mutated genes or abnormal molecules in tumors is of great therapeutic significance. Yet, no specific attentions have been made in this stage to cancer therapy PG. This is the most important shortcoming of cancer therapy PG.

Neoplasm metastasis treatment is different from primary tumor treatment [8–10,12–16]. Advanced cancer patients (with formed metastasizing colony remote from primary tumor) have poor survival outcomes. Similarly, cancer stem cell therapy PG also plays key roles for treatment failure, relapse and remote metastasis of cancer [17–22]. Genetic polymorphisms and genotyping of cancer metastasis-related or cancer stem cell-related genes or pathways might be very helpful for predicting drug responses to cancer tissues in patients with or without metastasis in future. This unique PG system nonetheless has very few new approaches and clinical successes.

### Prediction of drug resistance or not

One of the thorniest problems in clinical cancer trials is the occurrence of multidrug resistance (MDR) in tumor cells or tissues. Soon after cancer chemotherapy, a series of drug transporter or DNA repair molecules, such as ATP-binding cassette transporters (ABC transporters), p-glycoprotein (ABCB1, MDR1), MDR-related proteins (MRPs) and so on, work together to dramatically offset therapeutic efficacies and decide the nature of drug resistances and therapeutic failure in individual cancer patients. Thus, detecting polymorphisms of these genes and relevant biological molecules helps to predict the occurrence of drug resistance in tested cancer patients [8–10].

## Current cancer therapy PG routines & future trends

### The norm of prediction of drug responses

Predictions of drug responses against cancers and toxicity to the human body are indispensable parts of cancer therapy. The polymorphisms or epigenetic information of drug-targeted genes, tumor environmental molecules, drug metabolic enzymes, tumor suppressive genes, metastatic-related or cancer stem cell-related molecules can impact on the chemotherapeutic outcomes for cancer patients [3–10]. Proper handling or detection of genetic or epigenetic information of drug response genes and molecules are the key issues for individual cancer therapy.

### Diverse properties of cancer therapy PG

Anticancer PG and PG for other disease therapies generally share the same analytical routines and technical supporting systems (mostly single nucleotide polymorphisms [SNPs]) of drug-related or DME genes – the same as for other disease therapies. Since the DME genes for anticancer drugs are biologically identical in human bodies and regarded as parallel systems, no difference has been applied between anticancer therapy and other disease therapies in general hospital protocols and routines. Despite the homogeneity of human metabolic enzymes, etiologic and pathogenic processes between cancer and other diseases are diversified greatly. For example, the etiologies of most diseases initiate in a fixed string of genes and molecules – pathogens commonly come from outside infections. Moreover, drug targets for most disease categories are fixed and can be repeated again and again. Cancer drug responsiveness prediction is, nonetheless, different because cancer comprises different diseases with pathogenesis of unlimited growth and metastasis. The great diversity and unresolved mechanisms of action for cancer progression and proliferative or metastatic inhibitions make current cancer therapy PG at its initial stages. Much effort must be created and verified for cancer therapy PG improvements that can save the life of a great numbers.

### Great importance of ADME for anticancer prodrugs

Anticancer drugs, especially cytotoxic anticancer drugs, are highly toxic. Most cytotoxic anticancer drugs are even carcinogenic and can cause secondary tumors after administrations of higher than tolerated drug dosages. Approximately 1–10% of normal humans are deficient in one or several wild-type human metabolizing enzymes from one or several genetic polymorphisms. These patients cannot transform anticancer prodrugs into active metabolites (low-active drug components and responses) or reduce active metabolite clearance rates that lead to higher active metabolite concentrations in human plasma and toxicity. Increasing active anticancer drug or metabolite concentrations in patients’ plasma or normal tissues means significantly harmful impacts on cancer patient treatments, or even the cost of a patient's life [4,8–10] (Figure 1). Yet increasing concentrations or doses of therapeutic drugs for other diseases generally mean additions of side effects because side effects of most drug categories must be slight, moderate and hardly life-threatening. A high proportion of licensed anticancer drugs are made up of prodrugs and cytotoxic anticancer drugs. As a result, cancer therapy PG must be seriously investigated and applied in clinics, especially for prodrugs or cytotoxic anticancer drug treatments.

Different from other types of diseases, cancer is caused by a wide diversity of oncogenic mutations and environmental matrix and factors (such as vasculature activators like EGF or VEGF etc.) that support the transformations of normal cells into malignant ones [23]. More seriously, great heterogeneity occurs in same patients with malignant solid tumor categories, such as lung and breast cancer and so on [24]. Diversities of cancer genetic abnormality and molecular malfunctions require different categories of anticancer drugs or even anticancer drug combinations [8–10]. Approximately 84 anticancer or anti-metastatic drugs have been licensed in the USA [25] and 178 anticancer drugs are clinically utilized as of 2013 worldwide [26]. Meanwhile, anticancer drugs are mechanically diversified and tailored to different types of tumor categories. This unique characteristic of cancer and drug therapeutics is the major weakness of present PG routines of cancer therapy. More than 100 human genes might be involved in different types of human tumor categories and origin. Promoting scientific studies of the PG system for diversity of tumor origins and neoplasm metastasis is of great therapeutic importance.

## Possible solutions

Tumor growth and metastasis are determined by different cancer genes or hallmarks. Neoplasm metastasis is caused by the interactions of cancer cells and human environments [12]. Theoretically, a good therapeutic modular and clinical paradigm must be based on deep understanding of the relationship between pathology, pharmacology and therapy (next-generation cancer therapy PG if possible). Yet only small parts of cancer categories are well designed and tailored for advanced cancer patients. This leads to relatively poor predictive values of cancer therapy PG in clinics, especially patients with metastatic cancer. However, more than 90% cancer deaths are caused by cancer metastasis and cancer stem cells [12–16]. This is a problem of cancer biology rather than a problem of PG now. However, the two problems can be interrelated and borrow achievements from each other. Cancer therapy PG cannot become successful without completely understanding the relationship between cancer pathology and drug responses. The therapeutic efficacies against primary cancer growth and metastasis are not parallel [15,16]. More importantly, even the therapeutic efficacy of antimetastatic drugs is varied between different stages of cancer patients [15,16]. Cancer stem cells also lead to treatment resistance and cancer relapse [17–22]. Furthermore, anticancer drug combinations are widely used in clinical trials worldwide [8–10]. Nowadays, despite being a well-known strategy, the general anticancer drug combination PG systems have been unfinished in clinics, let alone relating to neoplasm metastasis and cancer stem cells.

## Dilemma for anticancer therapy PG

After the advent of cancer therapy PG, we have never attempted any new initiatives outside the boundary of the PG norm and technical routines that are different from widely practiced PG protocol in cancer therapy PG. If we stay on this course and in these mindsets, we might never have the opportunity to overcome the drawbacks and pitfalls of current cancer therapy and make a difference to now. Creative ideas and novel PG techniques for improving in cancer therapy are welcomed.

### A fixed cluster of dysfunction genes vs diversity backgrounds of gene mutations & alterations

Human cancer is a unique type of disease that in which a great diversity backgrounds of gene mutations and malfunctioning biomolecular profiling are found. According to general PG mindsets and routines, beside ADME-related metabolizing enzymes (≈300), at least the same amount of oncogenic genes can be mutated. From our past experience, at least 5–10 oncogenic genes or biomolecules will normally be found in tumor tissues among advanced cancer patients. Since a huge amount of human genes are related to cancer growth, metastasis, drug toxicities/responses, an indispensable topic is to design a high efficient strategy that can solve this technical difficulty. Brand-new strategies relevant to human gene and therapeutic prediction, even outside of current available polymorphisms information, might help us realize our dreams of making a difference to PG systems. However, this interesting topic seems unlikely to be solved by conventional PG routines in utilities.

### Requirements of effective PG models for anticancer drug combination predictions

Since cancer is a complicated disease, conventional cancer therapy needs more than one anticancer drug. As a result, anticancer drug therapy PG is different from other PG diseases. These differences for complex therapeutic recipes call for new ideas and PG routines. Obviously, the next generations of anticancer therapy PG systems must be capable of predicting complicated clinical circumstances and situations in clinical cancer trials. How can this goal be realized? It needs to build the technical capability of translating biological modular into therapeutic optimizing paradigms. Technical innovations and computational data analytical systems can guide us into new clinical horizon and predict complex formulae of cancer treatment recipes.

### PG of anticancer drug targets

Apart from genetics of metabolic enzymes, anticancer drug targeting genes and oncogenic- or metastatic-related genes are other parts of personalized cancer therapy [8–10,27–36]. Anticancer drugs exhibit anticancer activity by inhibiting targeted cancer genes or molecules. These targeted cancer genes or molecules are greatly diversified, and can be oncogenic, metastatic-related or cancer stem cell-related and so on [23]. More importantly, different anticancer drugs target different genes or molecules of cancer, such as complex ranges of genes, requiring building complicated PG systems in clinics.

### Possible PG therapeutic breakthrough for cancer therapy

Despite great monetary support and quick technical improvements, cancers remain to be an unresolved enigma and of therapeutic significance. Development of drug resistance, drug-induced severe side effects, cancer stem cells and tumor metastasis contribute to the majority of therapeutic failures in clinical cancer trials. Due to the poor drug specificity of many cytotoxic drugs, normal tissues are also damaged by such cancer treatments. As a result, the highest tolerated drug dosages cannot kill all tumor populations of large tumor volumes. Induction of drug resistance after conventional trials, cancer stem cells (mystery characteristics of tumors) and neoplasm metastasis and so on are the real culprits for therapeutic failures. Those PG systems designed to analyze genetic status of tumor or human tissues should be omnipotent for predicting both toxicity and efficacy of drugs in individual cancer patients. The present anticancer therapy PG systems are proving too little and too incapable for advanced cancer patients.

Since oncogenic- or metastatic-related genes or molecules are too diversified and disconnected, it is hard to find optimal drugs utilizing present PG systems and routines. Apart from PG systems, other personalized cancer therapy strategies such as drug sensitivity testing (DST) [8–10] or cancer bioinformatics [37,38] might be superior for drug choices and at ease to predict targeted genetic or molecular changes.

### New challenges

The overall theme of PG is the right drug for the right patient, by analyzing human genetics variations that play roles on predicting drug toxicities or responses. However, current PG systems are more suitable for fixed small ranges of pathological processes and change slightly after its advent. New knowledge coming from clinical case reports, doctors’ experience or hypothesis-driven systematic studies ought to provide a useful foundation for updating PG systems, especially cancer therapeutic PG systems and routines. After accumulating enough clinical data and large-scale human genome drafting, computation and validating, framing different genetic markers and pathogenic-related therapeutic efficacies are followed. Finally, we need to transform our knowledge and understanding from empirical into successful clinical paradigms [3–11]. Possible avenues are given. The current diversity of genes or molecules for PG study and clinical testing is tabulated and diagrammed below (Table 1 & Figure 2) [23].

Since genetic variations between individuals and disease progressions are not negligible, relationships between individual genes and drug therapeutic outcome are the priority. Much supportive information on this issue can easily come from other cancer biological or therapeutic advancements. Also, technical innovations can be implemented after absorptions of new fruits of scientific discoveries. However, basic rules behind the scenes are not understood yet.

### Genetic variations across different races or ethnic groups

An approximately 1–5% difference between different races or ethnic groups is present worldwide. Presently, PG studies are heavily reliant upon European descendants (Caucasian) [31,39]. These genetic makeup differences among different human races and ethnic groups might be suitable for different microarray chips or specific persons in clinical cancer trials [39]. How to design a new system that can quickly differentiate genetic information among all possible race and ethnic populations is the hot topic of the present and future.

### New PG systems suitable for every anticancer drug & drug combination

Determining the toxicities, activities and blood concentrations of anticancer drugs by PG plays an important role in clinical cancer trials. In spite of these utilizations, PG is not very useful for choosing the most suitable anticancer drugs from the large anticancer drug arsenal (≈84 anticancer agents or drugs licensed in the USA) [25] and 178 anticancer drugs licensed worldwide [26] (let alone huge numbers of drug combination possibilities). Long before, it has been found that drug combinations can commonly increase therapeutic efficacies and outcome in clinical cancer trials. Nevertheless, drug combinations will increase the complexity of pharmacokinetic detections and PG applications. Even though PG studies have been widely reported and reviewed [27–36], PG techniques have a long way to improve. In many clinical trials, drug plasma concentrations determined by modern chromatography are more straightforward than PG applications at this stage of PCT. Presently or in the future, we simply need to transform many new techniques from bench to the bedside by continuously perfecting PG routines and systems in modernized hospitals.

### Two ways to predict drug therapeutic efficacy in the general PG system

Two cancer therapy PG systems (drug-oriented PG and pathogenesis-oriented PG) have been categorized (Figures 3 & 4). Optimizing new PG systems by combinational or integrating both systems is much needed.

Moreover, potential new PG systems need an ingenious design via integration of two systems and scientific investigations supporting improvement of PG routines.

Presently, cancer therapy PG, however, is only superior for drug dosages or toxicity determinations by SNPs of drug ADME profiling.

An imbalance between the rapid development of genotyping technology and the slow pace of genetic testing marketing is generally met. The great degree of uncertainty in interpreting drug responses to tumor progressions, stem cells or metastasis PG has not been the breakthrough in cancer therapy worldwide. Despite a lot of successful stories, presently cancer therapy PG does not develop into compulsory routines, even in developed countries. A shortage of large sample-sized retrospective or clinical cohort studies has been reported [39]. Moreover, pharmaceutical companies also do not enthuse supporting of PG work in every new drug development [39]. In order to promote and transform PG testing into clinical mandatory applications, PG progresses based on worldwide cooperation among drug manufacture, academic and government-funding bodies should not be overlooked. Using this argument, we must accumulate enough data of genotype–phenotype association, genetic–drug relationship and genome-wide association study (GWAS) information for the reality of increasing cancer patients’ survivals by PG innovations.

### Genome-wide association studies

GWAS are always the priority for PG technical updating. Owing to the invention of next-generation sequencing (NGS) techniques, human genome sequencing can be a joint effort between biomedical students and mathematical or physics students or scholars in the future. For these undertakings, mathematics or physics students and scholars will prove to be more adept than biomedical students in the postgenomic study age. Since the tremendous speed-up (5000- to 50,000-times) and low budget (US$7,500 per genome) of genotyping human genomes by NGS [40–43] compared with genotyping systems in the era of the human genome projects from 1990 to 2000 (US$3 billion), GWAS for promoting cancer therapy PG or other types of personalized cancer therapies will soon become a reality.

Bioinformatics (omics technology) are more advantageous for quantifying specific gene or biological molecular changes than those used by current PG systems. At first, we need to detect the clinical data of both PG and bioinformatics. Then, ingeniously designing a new generation of PG techniques or systems that can provide genetic information of both quantity and quality, even omnipotent systems, are possible routes to renew PG techniques for cancer therapy predictions. Cooperating merits from NGS and GWAS or other new techniques may improve cancer therapy PG or even change the landscape of personalized cancer therapies worldwide. Then, improvements of predicting therapeutic efficacies, toxicity and outcomes may be realized by integrating growing bodies of diagnostic or pathological profile information.

### Cost-effective considerations

Anticancer drug responses in tumor tissues and human bodies, especially those of cytotoxic anticancer drugs, are often multigenetic and multifactorial [44,45]. Presently, complexity means PG of anticancer or combinative therapies is beyond our reach. Therefore, cancer therapy PG has much room to improve and costs for PG testing of one patient (generally US$30–5000) make them unpractical for worldwide uses at present [46–48]. However, comparing that with the cost of anticancer drugs or hospital residence fees in developed countries, cancer therapy PG is cost-effective [46]. In the future, might it be possible to pay the PG fee considering the treatment outcomes in clinical cancer trials [48]?

## Conclusion

No central dogma or paradigms of cancer therapy PG are capable of being universally utilized. Borrowing ideas and lessons from other scientific disciplines, hypothesis-driven data collections and workable computations for revealing the relationship of cancers diversity and a variety of therapeutic options are important resources for inventions of new generations of PG systems. In future, cancer therapy PG might transform from a number of genetic testing modalities into omnipotent, science-guarded and high-throughput predictive systems. The summary table outlines the roadmap and avenues of future directions of cancer therapy PG past and in future.

Briefly, PG in clinics might no longer be considered a hobby in the future. Increasing occurrences of mandatory PG trials might be required in most advanced countries, or even become indispensable worldwide.

## Future perspective

Since we speculate that key breakthroughs of anticancer PG may not come from simply increasing the sample-size of clinical PG data, it relies on injections of insights and breakthrough of other disciplines, biological/medical discoveries or developments of new generations of anticancer drugs [49–55] and rules of drug combinations (Figure 5).

Present cancer therapy PG systems are imperfect for large populations of cancer deaths (7–10 millions annual cancer deaths worldwide). Possible future cancer therapy PG advancements may come from ideas and outcomes of other researchers instead of keeping up present mindsets and hospital routines.

**Table 1.** **Mechanism categories of anticancer drug pharmacogenetics.**

**Major biological types**	**Gene targets**
Cancer stem cell-related genes or molecules	β-catenin; TGF-β; SDF1-CXCR4-CXCL12; MDR transporter
Downstream mechanisms	Apoptosis genes; chemokines; p53 (drug response or resistance; Bcl, FAS/CD95/APO-1, PTEN, TNFs and IL-10); IL-6
Drug–target interactions	DNA metabolism and biosynthesis (alkalating agents and platinum drugs); DNA repair mechanisms (toxicity or resistance of cytotoxic anticancer drugs); cell signal receptors’ mitotic spindle (possibility of drug resistance); hormonal-regulated enzyme; HIF-related pathways; nuclear factor-related pathway
Tumor metastasis-related genes or pathways	Angiogenesis genes; cadherin; cell adherin molecules; integrin; metal matrix proteinases; selectin; sialic acid-related genes

CAM: Cell adherin molecule; MMP: Metal matrix proteinase.

**Executive summary**

**Previous highlights**

Techniques and strategies for prediction of both toxicity and efficacy in individual cancer patients.
Pharmacogenetics (PG) study, especially drug absorption, distribution, metabolism and excretion molecules, such as metabolic enzyme genes and dose-optimizing.
Description of established PG protocols and routines, comparisons between cancer therapy and other disease therapies.
Outlook of different categories of PG techniques, such as drug-targets and disease-based genetic systems.
Discover and predict the relationship between cancer pathology and treatments by PG practice in individual cancer patients.
Find ways of pinpointing the diversity of pathologic origin and different therapy of primary, stem or metastatic cells or tissues in each cancer patient and be able to maximize the efficacy of drugs or therapy.

**New challenges**

Lacking established relations between therapeutic outcomes and PG applications.
Limitations and shortcomings of conventional PG techniques and systems.
The importance of neoplasm metastasis, multidrug resistance and cancer stem cells in cancer therapeutic outcome improvements.
Comparisons of therapeutic efficacies between primary tumors and metastatic nodules.

**Conclusion & future perspective**

Create some original, innovative systems or biomedical software for integrating diversified and combinative pathological, pharmacological and clinical information into utility paradigms.
Invest more money into developments of effective antimetastatic drugs or cancer stem cell inhibitors.
Construct usable anticancer drug combinative PG systems.
Promote genome-wide association studies, especially among different ethnic groups, races and/or tumor types and stages.
